# Author Correction: An anti-influenza A virus microbial metabolite acts by degrading viral endonuclease PA

**DOI:** 10.1038/s41467-023-39437-x

**Published:** 2023-06-23

**Authors:** Jianyuan Zhao, Jing Wang, Xu Pang, Zhenlong Liu, Quanjie Li, Dongrong Yi, Yongxin Zhang, Xiaomei Fang, Tao Zhang, Rui Zhou, Zhe Guo, Wancang Liu, Xiaoyu Li, Chen Liang, Tao Deng, Fei Guo, Liyan Yu, Shan Cen

**Affiliations:** 1 Institute of Medicinal Biotechnology, Chinese Academy of Medical Sciences and Peking Union Medical School, 100050 Beijing, PR China; 2 Lady Davis Institute for Medical Research, Jewish General Hospital, McGill University, Montreal, H3T 1E2 Canada; 3 Institute of Microbiology, Chinese Academy of Sciences, 100101 Beijing, China; 4 Institute of Pathogen Biology, Chinese Academy of Medical Sciences and Peking Union Medical School, 100730 Beijing, PR China

**Keywords:** Antiviral agents, Influenza virus, Target identification, Post-translational modifications

Correction to: *Nature Communications* 10.1038/s41467-022-29690-x, published online 19 April 2022

The original version of this Article contained errors in Figure 6 and its corresponding legend; Supplementary Table 2; the Source Data; Methods; and the competing interests statement.

**Figure 6**

The original version of Figures 6a, 6b, and 6c incorrectly reported that mouse survival, weight, and influenza titers in lung tissue samples, respectively, were tracked up to 13 days post-infection; the original Y-axis of Figure 6c was incorrectly labelled as reporting PFU.

Figures 6a, 6b, and 6c have been respectively corrected to report data on mice survival, weight changes, and influenza titers in lung tissue up to days 14 post-infection. The Y-axis of Figure 6c is now correctly labelled as log10 TCID80/0.1ml.

**Figure 6 legend**

The legend of Figure 6 has been corrected to:

“Mice (six per group) were infected with IAV WSN/33 (5 × LD50, in 50 μL PBS), and then treated with either APL-16-5 (4, 20, or 100 mg/kg), ribavirin (100 mg/kg), or PBS daily from day 1 to 8 post-infection. **a** Survival rate (*n* = 6), **b** body weight (*n* = 6). In a separate animal study of the same designs, **c** viral titers (TCID_50_) in the lungs (*n* = 3) and **d** histopathology (one out of three shown, 200 X) were determined. Bar = mean. Error bars=±SEM. For (**c**) viral titers at 4 and 14 DPI or until the death of mice in PBS-treated group (PBS-treated vs. Ribavirin, APL-16-5-100, 20, 4 mg/kg): (*p* = 0.0029, *p* = 0.0006, *p* = 0.0015 and *p* = 0.0015, *p* = 0.0001, *p* = 0.0001, *p* = 0.0016, and *p* = 0.0015, respectively), an unpaired two-tailed t-test was used. **p <  0.01, ***p < 0.001. Source data are provided as a Source Data file”.

**Reporting of results shown in Figure 6**

The Results have been revised to correctly describe (I) the length of the experiments, (II) the description of the different mouse cohorts in each individual experiment, and (III) the procedure for determining viral titers from lung tissue. Accordingly, the Source Data for Figure 6, as well as the summary of the data that is presented in Supplementary Table 2 have been updated to reflect the correct number of animals included in each experiment.

In the Results sub-section entitled, “APL-16-5 protects mice from lethal IAV infection”, we have corrected the description of the results of the influenza viral infection experiments to:

*“*Remarkably, 100% of the mice treated with 100 mg/kg APL-16-5 and 67% of mice treated with a low dose of APL-16-5 (4 mg/kg) survived IAV infection, while none of the mice treated with PBS survived (Fig. 6a). APL-16-5 treatment increased the survival of mice, reaching 75% (Supplementary Table 2)” and “Moreover, viral titers in the lungs were significantly reduced on days 4 and 14 post-infection in mice treated with APL-16-5, especially at doses of 20 and 100 mg/kg (Fig. 6c)*”* respectively.

**Methods**

In the sub-section of the Methods entitled, “IAV infection of mice”, the description of the influenza inoculation experiments, and analyses of the results has been amended to read:

“Mice (six per group) were intranasally infected with influenza A/WSN/33 virus (5 × LD50, in 50 μL PBS) or mock-infected, and then received either APL-16-5 (4, 20, or 100 mg/kg), ribavirin (100 mg/kg), or PBS daily on days 1–8 post-infection. Compound diluent was 0.5% Sodium Carboxymethylcellulose. Mice were monitored daily, and survival and weight loss were recorded up to day 14. In a separate animal study of the same designs, virus titers in the lungs were determined from mice (three mice from each group) sacrificed on day 4 and 14 post virus exposure or until animal died. Mouse lung was collected and homogenized. Each sample was assayed in triplicate for viral titers in MDCK cells with the TCID_50_ method. Samples for histopathology examination were collected on day 14 after virus inoculation or until animal died. The lungs were fixed in 4% paraformaldehyde”.

Additionally, we have now added a sub-section for our ZIKV infectivity assay into the Methods. “Titer of ZIKV stock was determined by infecting Vero cells, the results were calculated as the median tissue culture infective dose (TCID_50_). Vero cells were infected with ZIKV at an MOI of 0.01. ZIKV infection was determined by quantifying viral RNA with qRT-PCR. Total cellular RNA was extracted 48 h post-infection with TRIzol reagent (Invitrogen, USA). cDNA was synthesized using Primescript RT Master Kit (Takara, Japan). Level of viral RNA was determined by qRT-PCR using SYBR premix Ex Taq II kit (Takara). ZIKV gene NS2A was amplified with primers 5′-CCACGCACTGATAACAT-3′ (forward) and 5′-AAGTAGCAAGGCCTGCTCT-3′ (reverse). Cellular GAPDH RNA was amplified with primer pair 5′-atcatccctgcctctactgg-3′/5′-gtcaggtccaccactgacac-3, the results served as internal controls to normalize ZIKV RNA data. For quantification, the 2−ΔΔCt method was used to calculate the relative ZIKV RNA levels against GAPDH”.

**Source Data**

The original Source Data associated with Supplementary Figure 2c contained a number of copy and paste errors. The treatment groups, ‘G9-2’ and ‘G9-10’ have been corrected to ‘APL-16-5-(2 μM)’ and ‘APL-16-5-(10 μM)’, respectively. One additional copy and paste errors for Replicates 1 and 2 in the ‘APL-16-5-(2 μM)’ group have been corrected; for Replicate 1 the reported Ct value of 22.103 has been corrected to 22.091, and for Replicate 2 the reported Ct value of 22.584 has been corrected to 22.576.

**Competing Interests**

The competing interests statement has been updated in the revised article to include patent information. The statement now reads: ‘S.C., L.Y., J.Z., Y.Z., T.Z., X.P. and X.F. are inventors of the related patent/application 201710077812.X., which was filed by the Institute of Medicinal Biotechnology. This patent includes some in vivo antiviral data described herein, which does not impose any restrictions for the scientific use of the related compounds. The remaining authors declare no competing interests’.

These corrections do not affect the main conclusions of the study. The PDF and HTML versions of the Article have been updated.

## Supplementary information

Updated Supplementary Information

## Source data

Source Data

