# Supplementary material for: An anti-influenza A virus microbial metabolite acts by degrading viral endonuclease PA
Source: Nat Commun. 2022 Apr 19;13:2079. doi: 10.1038/s41467-022-29690-x (PMC9019042; doi:10.1038/s41467-022-29690-x)
Supplement: Supplementary file 1 — Supplementary Information [file 41467_2022_29690_MOESM1_ESM.pdf]

Supplementary Information File

An anti-influenza A virus microbial metabolite acts by degrading viral endonuclease PA

Jianyuan Zhao, Jing Wang, Xu Pang, Zhenlong Liu, Quanjie Li, Dongrong Yi, Yongxin Zhang, Xiaomei Fang, Tao Zhang, Rui Zhou, Tao Zhang, Zhe Guo, Wancang Liu, Xiaoyu Li, Chen Liang, Tao Deng, Fei Guo, Liyan Yu, Shan Cen

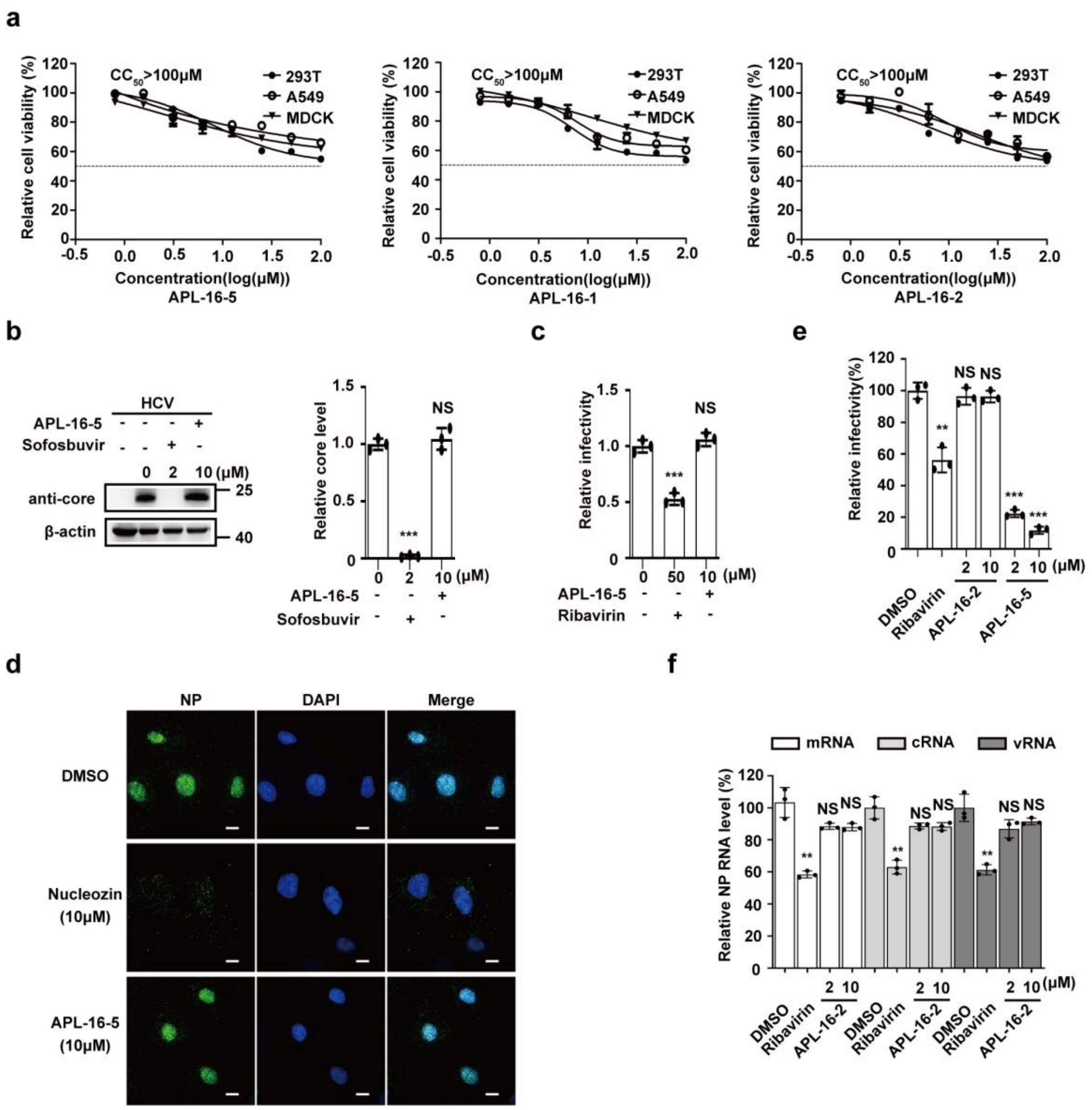

Supplementary Fig. 1 Identification of APL-16-5 as a potent influenza A virus (IAV) inhibitor by targeting *de novo* viral replication a, Cytotoxicity of APL-16-5, APL-16-1, and APL-16-2 was measured by CCK-8 assay in HEK293T, A549, and MDCK cells. **b**, Huh7.5.1 cells were infected with HCV JFH1 strain at an MOI of 0.01, and incubated with APL-16-5 at 10 μM. Sofosbuvir was used as a positive control. Seventy-two-hours after infection, viral core protein and cellular β-actin expression were determined by western blotting. Protein band intensity was determined using the ImageJ program. Bar =mean. Error bars=±SEM. For DMSO vs. sofosbuvir and APL-16-5: (p =0.0001 and p =0.4935, respectively), an unpaired two-tailed t-test was used. ***p<0.001, NS=not significant. **c**, qRT-PCR was used to quantify viral RNA in APL-16-5 (10 μM)-treated Vero cells infected with ZIKV at an MOI of 0.01. Bar =mean. Error bars=±SEM. For DMSO vs. Ribavirin and APL-16-5: (p =0.0004 and p =0.3890, respectively), an unpaired two-tailed t-test was used. ***p<0.001, NS=not significant. **d**, Immunofluorescence was performed to detect the nuclear import of IAV RNP in the presence of APL-16-5. Nucleozin was used as a positive control. Samples were stained for nuclei (blue) and viral NPs (green). The scale bars represent 5 μm. **e**, HEK293T cells were infected with single-round infectious IAV at an MOI of 0.2, in the presence of APL-16-5. Gluc activity was measured. Ribavirin (30 μM) was used as a positive control. Bar =mean. Error bars=±SEM. For DMSO vs. Ribavirin, APL-16-2-2, 10 μM, APL-16-5-2, 10 μM): (p =0.0013, p =0.4536, p =0.3673, p =0.0001, p =0.0001, respectively), an unpaired two-tailed t-test was used. **p<0.01, ***p<0.001, NS=not significant. **f**, qRT-PCR to

quantify viral RNA (mRNA, vRNA, cRNA) in WSN/33-infected A549 cells treated with various amounts of APL-16-2. Bar =mean. Error bars=±SEM. For NP mRNA, cRNA, vRNA (DMSO vs. Ribavirin, APL-16-2-2, 10 μM): ($p = 0.0013$, $p = 0.0540$ and $p = 0.0504$, $p = 0.0015$, $p = 0.0530$ and $p = 0.0525$, $p = 0.0018$, $p = 0.0914$ and $p = 0.1679$, respectively), an unpaired two-tailed t-test was used. **$p<0.01$, NS=not significant. Source data are provided as a Source Data file.

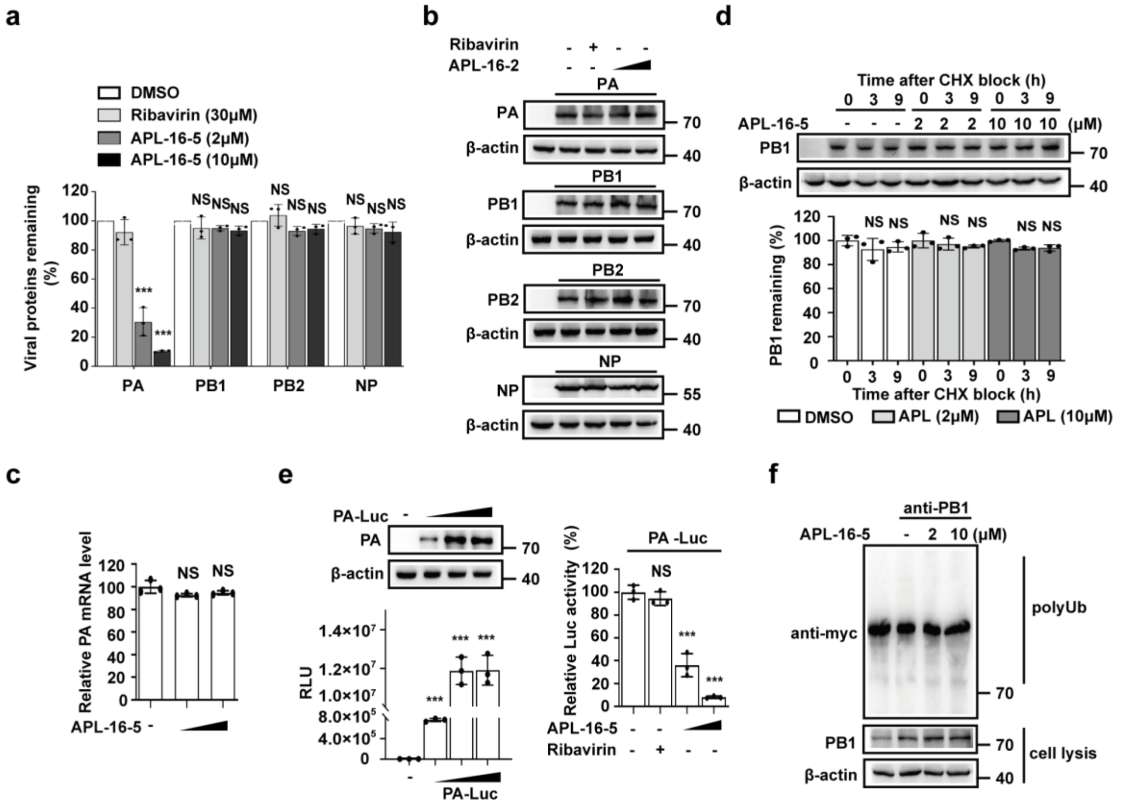

Supplementary Fig. 2 APL-16-5 induces proteasome-dependent PA degradation. **a**, Quantification analysis of western blot of Fig. 2b. For PA, PB1, PB2, NP (DMSO) vs. PA, PB1, PB2, NP (Ribavirin-30μM, APL-16-5-2μM, APL-16-5-10μM): (p =0.5546, p=0.0057 and p =0.0002, p =0.2471, p=0.1553 and p =0.0695, p =0.9609, p=0.1034 and p =0.1069, p =0.3182, p=0.2276 and p =0.1859, respectively). **b**, Western blot analysis of lysates from HEK293T cells transfected with plasmid PA and treated with DMSO or APL-16-2 (2 μM or 10 μM). **c**, qRT-PCR was used to quantify PA mRNA in cells transfected with PA DNA and treated with various concentrations of APL-16-5 (2 μM or 10 μM). For DMSO vs. APL-16-5-2, 10 μM: (p =0.1564 and p =0.3079, respectively). **d**, Western blot analysis of lysates from HEK293T cells transfected with plasmid PB1 in the presence of APL-16-5 for the indicated time intervals. For DMSO, APL-16-5-2, 10 μM (0h) vs.DMSO, APL-16-5-2,10 μM (3h and 9h): (p =0.7311 and p=0.1044, p =0.9241 and p =0.8942, p =0.4096 and p =0.2550, respectively). **e**, HEK293T cells were transfected with plasmid PA-luc (50, 100, 200 ng) for 24 h. Luciferase activity was measured to determine the PA level. For PA-luc (0 ng vs. 50 ng, 100 ng, 200 ng): (p =0.0001, p=0.0001 and p =0.0001, respectively). Western blotting was performed to determine the levels of viral PA and cellular β-actin (left). HEK293T cells were transfected with the PA-luc plasmid in the presence of APL-16-5 (2 μM or 10 μM), and luciferase activity was measured to quantify PA levels (right). For PA-luc (DMSO vs. Ribavirin, APL-16-5-2, 10 μM): (p =0.3371, p=0.0007 and p =0.0001, respectively). **f**, Immunoprecipitation and immunoblot analysis of HEK293T cells transfected with PB1 and CW7-myc-ubiquitin DNA in the presence of increasing amounts of APL-16-5. Lysates were subjected to immunoprecipitation with an anti-PB1 antibody and immunoblotted with an anti-Myc antibody. (**a, c**-**e**) The graph summarizes n = 3 independent experiments. Error bars show mean ± SEM (unpaired two-tailed t-test). ***p<0.001, NS=not significant. Source data are provided as a Source Data file.

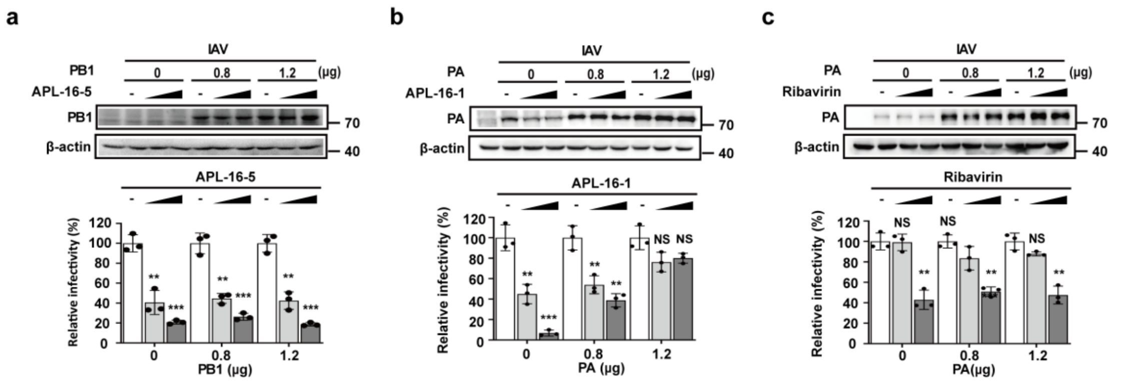

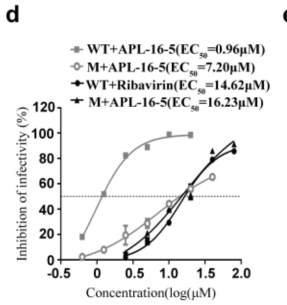

e

Mutations of PA in viruses resistant to APL-16-5

Samples	Sites of mutations													
	16	177	74	228	266	321	333	463	512	562	603	619	632	704/705
1			H74Y	N228K			N333D							AS704H.
2				N228K				V463A		F562L				AS704H.
3				N228K					R512G					AS704H.
4				N228K	R266T									AS704H.
5				N228K		N321D							S632P	AS704H.
6	L16I	T177A		N228K							K603R	W619.		AS704H.
7				N228K										AS704H.

Supplementary Fig. 3 APL-16-5 inhibits influenza A virus (IAV) by inducing PA degradation. a, b and c, HEK293T-Gluc cells were transfected with increasing concentrations of PB1 (**a**) or PA (**b, c**), followed by WSN/33 infection at a multiplicity of infection (MOI) of 0.5, and then treated with APL-16-5 (**a**), APL-16-1 (**b**) or ribavirin (**c**). Then Gluc activity was determined. Levels of PB1 (**a**) and PA (**b, c**) proteins were determined by western blotting. For (**a**) PB1 0, 0.8, 1.2 μg (DMSO vs. APL-16-5-2, 10 μM): (p =0.0023 and p =0.0001, p =0.0011 and p =0.0003, p =0.0030 and p =0.0001, respectively), for (**b**) PA 0, 0.8, 1.2 μg (DMSO vs. APL-16-1-2, 10 μM): (p =0.0038 and p =0.0002, p =0.0057 and p =0.0005, p =0.0528 and p =0.0514, respectively), for (**c**) PA 0 0.8, 1.2 μg (DMSO vs. Ribavirin-5, 20 μM): (p =0.8990 and p =0.0014, p =0.0971 and p =0.0018, p =0.0681 and p =0.0016, respectively. **d**, HEK293T-Gluc cells were infected with wild-type or PA mutant virus at a multiplicity of infection (MOI) of 0.5 and treated with APL-16-5 or ribavirin at the indicated concentrations for 24 h, then Gluc activity was determined. **e**, Summary of mutations in the PA protein based on the sequencing of APL-16-5-resistant viruses. (**a-c**) The graph summarizes n = 3 independent experiments. Error bars show mean ± SEM (unpaired two-tailed t-test). **p <0.01, ***p<0.001, NS=not significant. Source data are provided as a Source Data file.

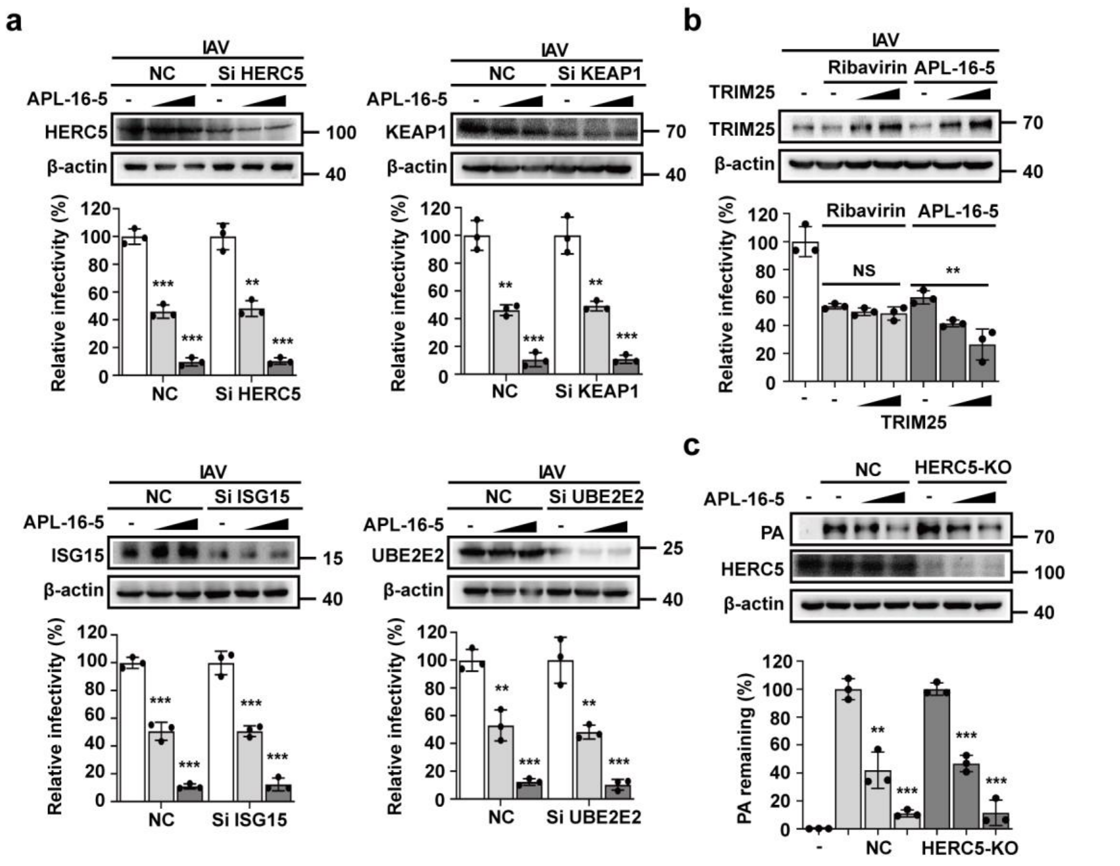

Supplementary Fig. 4 TRIM25 is required for APL-16-5-induced PA degradation. a, HEK293T-Gluc cells were transfected with HERC5, KEAP1, ISG15 and UBE2E2 siRNAs and infected with IAV WSN/33 in the presence of increasing concentrations of APL-16-5 (2 µM or 10 µM). Antiviral activity of APL-16-5 was determined by measuring Gluc activity. HERC5, KEAP1, ISG15 and UBE2E2 levels were measured by western blotting. For HERC5, KEAP1, IAG15, UBE2E2 NC (DMSO vs. APL-16-5-2, 10 µM): (p =0.0002 and p =0.0001, p =0.0012 and p =0.0002, p =0.0004 and p =0.0001,p =0.0040 and p =0.0001, respectively), HERC5, KEAP1, IAG15, UBE2E2 Si RNA (DMSO vs. APL-16-5-2, 10 µM): (p =0.0012 and p =0.0001, p =0.0030 and p =0.0003, p =0.0008 and p =0.0001, p =0.0067 and p =0.0008, respectively). **b**, Western blot analysis of lysates from HEK293T-Gluc cells transfected with increasing concentrations of TRIM25 (0.5 µg or 1.0 µg) in the presence of APL-16-5 (2 µM) or ribavirin (20 µM). Antiviral activity was determined by measuring Gluc activity. For Ribavirin, APL-16-5 (TRIM25 0 µg vs. 0.5 and 1.0 µg): (p =0.1062 and p =0.1528, p =0.0037 and p =0.0084, respectively). **c**, Western blot analysis of lysates from HERC5-knockdown HEK293T-Gluc cells transfected with PA DNA in the presence of increasing concentrations of APL-16-5 (2 µM or 10 µM). Antiviral activity of APL-16-5 was determined by measuring Gluc activity. For HERC5 NC, si RNA (DMSO vs. APL-16-5-2, 10 µM): (p =0.0028 and p =0.0001, p =0.0002 and p =0.0001, respectively). (**a-c**) The graph summarizes n = 3 independent experiments. Error bars show mean ± SEM (unpaired two-tailed t-test). **p <0.01, ***p<0.001, NS=not significant. Source data are provided as a Source Data file.

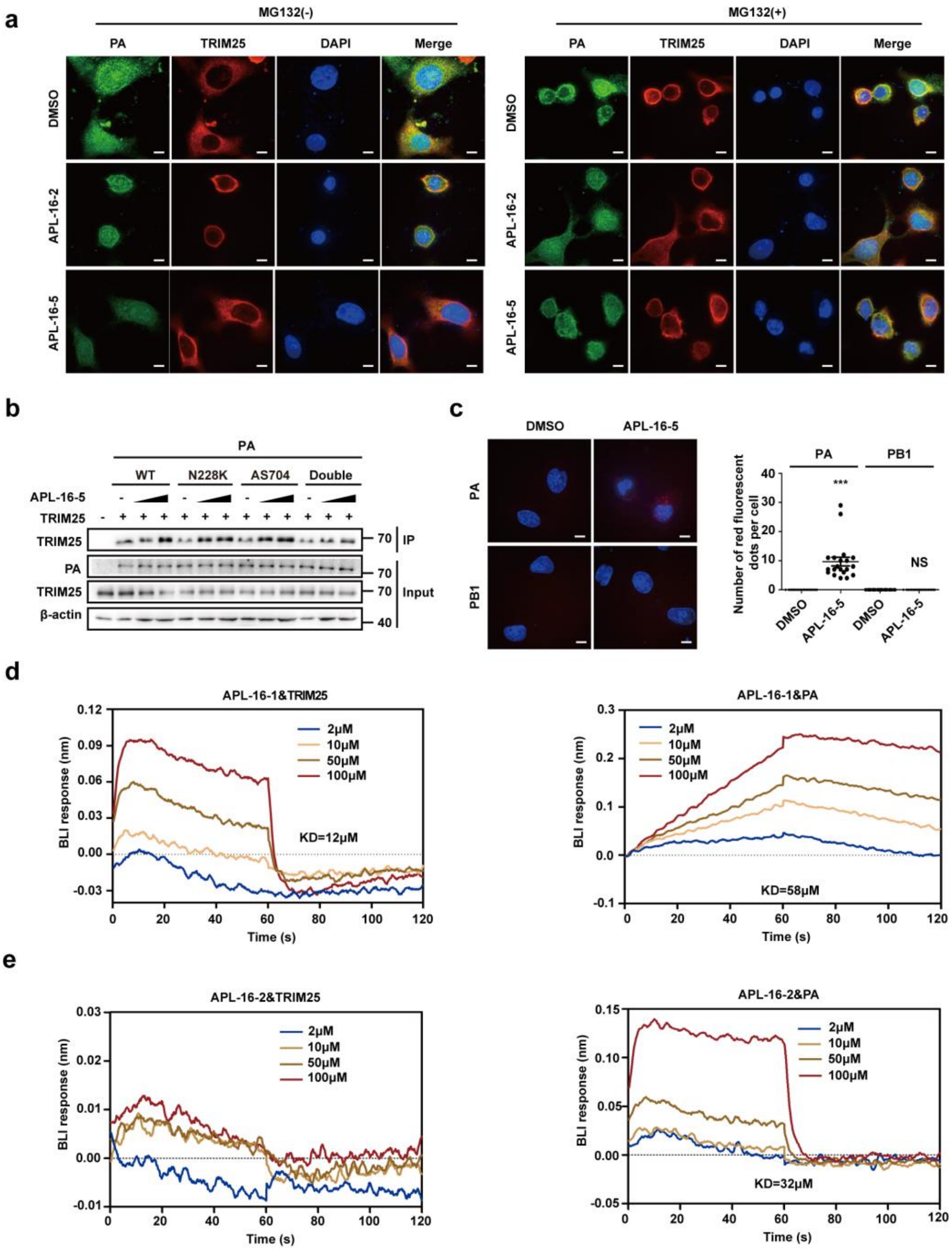

Supplementary Fig. 5 APL-16-5 causes TRIM25 to interact with, and ubiquitinate, viral PA protein. a, Confocal microscopy was used to evaluate the co-localization of TRIM25 (red) and PA (green) in the presence of APL-16-5 (10 μM) or APL-16-2 (10 μM) with or without MG-132 (5 μM). The scale bars represent 5 μm. **b**, HEK293T cells were transfected with wild-type or mutated PA DNA in the presence of increasing concentrations of APL-16-5 (2 μM or 10 μM) . Twenty-four-hours post transfection, lysates were subjected to IP with an anti-TRIM25 antibody and immunoblotted with an anti-PA antibody. **c**, *In situ* PLA was performed to detect the interaction between TRIM25 and PA in the presence of APL-16-5 (10 μM) using the HEK293T cell lines. One representative confocal images are shown. The scale bars represent 5 μm. (left). Quantification of PLA red fluorescence signals from 20 cells (right). Bar =mean. Error bars=±SEM. For PA, PB1 (DMSO vs. APL-16-5): (p =0.0001 and p =1, respectively) a paired two-tailed t-test was used. ***$p < 0.001$. NS = not significant. **d** and **e**, *In vitro* binding of APL-16-1 or APL-16-2 to purified TRIM25 (**d**) and PA (**e**) as determined by BLI binding assay. Representative sensorgrams are shown. Source data are provided as a Source Data file.

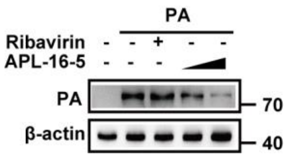

Supplementary Fig. 6 APL-16-5 degrades PA in BHK21 cells. BHK21 cells were transfected with plasmid PA and treated with DMSO or APL-16-5 (2 μM or 10 μM) for 24 h. Lysates were subjected to immunoblotting with a PA or β-actin antibody. Source data are provided as a Source Data file.

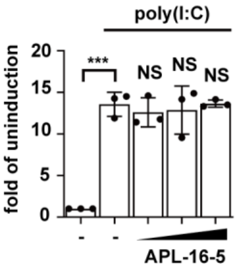

Supplementary Fig. 7 APL-16-5 does not affect IFN-β production in HEK293T cells. IFN-β (Luc) DNA was transfected into HEK293T cells. Twelve-hours post-transfection, cells were treated with poly (I:C) (1 µg/mL) in presence of APL-16-5 (0 µM, 5 µM, and 10 µM). Luciferase activity was measured at 24 h, for 24 h (n=3). Bar =mean. Error bars=±SEM. For poly(I:C) (-) vs. poly(I:C) (+): (p =0.0001), APL-16-5 (0 µM vs.5 and 10 µM): (p =0.5023, p=0.7277, p=0.9217, respectively) a unpaired two-tailed t-test was used. ***p < 0.001. NS = not significant. Source data are provided as a Source Data file.

Supplementary Table 1 Antiviral activity of APL-16-5 against different strains of influenza virus

Host cells	Virus strains	EC_{50} (μM)	
		APL-16-5	Ribavirin
A549	A/WSN/1933(H1N1)	0.67±0.11	22.62±1.78
	A/Puerto Rico/8/1934(H1N1)	0.86±0.12	25.21±1.23
	A/Beijing/30/95 (H3N2)	0.57±0.02	20.54±0.89
	B/Beijing-Haidian/1386/2013	1.45±0.07	26.67±1.34
	B/Massachusetts/2/2012	2.10±0.21	28.23±1.35
MDCK	A/WSN/1933(H1N1)	1.26±0.44	21.67±1.34
	A/Puerto Rico/8/1934(H1N1)	0.69±0.13	20.67±2.91
	A/Beijing/30/95 (H3N2)	0.96±0.03	24.78±1.09
	B/Beijing-Haidian/1386/2013	1.22±0.18	23.65±2.65
	B/Massachusetts/2/2012	4.16±0.38	20.31±3.21

Supplementary Table 2 APL-16-5 protects mice from lethal influenza A virus infection (X±s)

Group	Days	Life extension (%)
Normal	14	-
PBS-treated	8 ± 1.55	-
Ribavirin(100 mg/kg)	14***（p=0.0001）	75.00
APL-16-5(100 mg/kg)	14***(P=0.0001)	75.00
APL-16-5(20 mg/kg)	13.83±0.41***(P=0.0001)	72.92
APL-16-5 (4 mg/kg)	12.67 ± 2.16**(p=0.0016)	58.33

Supplementary Table 3 Lung index of APL-16-5 in influenza A virus-infected mice (X±s)

Groups	Lung index	Lung index inhibition (%)
Normal	0.63 ± 0.08	-
PBS-treated	0.93 ± 0.09	-
Ribavirin (100 mg/kg)	0.68 ± 0.05***(p=0.0001)	82.97
APL-16-5 (100 mg/kg)	0.69 ± 0.05***(p=0.0002)	79.45
APL-16-5 (20 mg/kg)	0.74 ± 0.08**(p=0.0031)	61.26
APL-16-5 (4 mg/kg)	0.81 ± 0.15	40.39

Supplementary Table 4 Protein profile of cells treated with APL-16-5 using a quantitative proteomics

Gene name	Fold change*	log2 fold change	P value	MW [kDa]	Coverage [%]	MS/MS Counts	Unique peptides
FN1	0.3189	-1.648823997	7.02384E-05	272.32	18.8	269	34
LAMB1	0.6532	-0.614403304	0.003985268	198.04	9.4	45	11
SLC1A3	0.6507	-0.619935541	0.014006929	59.572	7.9	19	3
GALNT1	0.6148	-0.70181093	0.001527578	64.218	24.7	54	11
PCLAF	0.5032	-0.990796173	0.006137757	11.986	45.9	18	4
PON2	0.6426	-0.638007114	0.023661548	39.38	27.1	65	7
RAB12	0.6438	-0.635315518	0.03839378	27.248	22.1	27	5
FAM117B	0.6579	-0.604059782	0.00150948	61.967	8.7	5	3
GOLM1	0.6296	-0.667492554	0.02675005	45.333	16.5	26	5
SHCBP1	0.6335	-0.658583476	0.020997328	75.69	25.4	51	13
TEFM	0.6327	-0.660406499	0.003313502	41.676	14.2	19	3
SDF4	0.4219	-1.245027007	0.001106222	41.806	31.5	56	8
RIOK2	0.6055	-0.723801135	0.003462745	63.282	26.3	51	8
RBM24	0.3778	-1.404305393	0.008330851	24.776	11	14	2
KLF16	0.5083	-0.976247864	0.00280039	25.43	23.8	25	3
ETNK1	0.6243	-0.679688629	0.004512983	50.968	13.5	33	4
ACOT13	0.5817	-0.781652791	0.011777189	14.96	52.9	27	4
SMYD2	0.6353	-0.654490076	0.022053778	49.688	14.3	18	4
NDUFB11	0.5662	-0.820616346	0.029472673	17.316	34.6	23	4
TMEM9	0.631	-0.66428809	0.024264699	20.574	32.8	20	3
WRAP73	0.6264	-0.674843882	0.003325938	51.588	20.7	26	6
MAD2L2	0.5688	-0.81400663	0.047251808	24.334	32.7	14	3

Note: * means APL-16-5 treated HEK293T / control HEK293T

Supplementary Table 5 Protein profile in the TRIM25 knockout cells treated with APL-16-5

Gene name	Fold change*	log2fold change	P value	MW [kDa]	Coverage [%]	MS/MS Counts	Unique peptides
FN1	2.1129	1.079224489	0.00057006	272.32	18.8	269	34
LAMB1	0.6988	-0.517048487	0.078023105	198.04	9.4	45	11
SLC1A3				59.572	7.9	19	3
GALNT1	1.1299	0.176195095	0.33929835	64.218	24.7	54	11
PCLAF				11.986	45.9	18	4
PON2	1.0094	0.013497992	0.898420518	39.38	27.1	65	7
RAB12	1.0347	0.049212535	0.717959892	27.248	22.1	27	5
FAM117B				61.967	8.7	5	3
GOLM1	0.4493	-1.154249033	0.002472191	45.333	16.5	26	5
SHCBP1	1.4119	0.497637911	0.000601133	75.69	25.4	51	13
TEFM	0.9127	-0.131787364	0.904167113	41.676	14.2	19	3
SDF4	0.2909	-1.781404798	0.000173843	41.806	31.5	56	8
RIOK2	1.21	0.275007047	0.023858405	63.282	26.3	51	8
RBM24	1.868	0.901494455		24.776	11	14	2
KLF16	0.9068	-0.141143704	0.513164405	25.43	23.8	25	3
ETNK1				50.968	13.5	33	4
ACOT13	1.0102	0.014640947	0.85684446	14.96	52.9	27	4
SMYD2	2.4679	1.303283937		49.688	14.3	18	4
NDUFB11	0.7173	-0.479351464	0.39918933	17.316	34.6	23	4
TMEM9	1.0511	0.071899932	0.63580078	20.574	32.8	20	3
WRAP73	1.2378	0.307778227	0.206161033	51.588	20.7	26	6
MAD2L2	0.8404	-0.250851933	0.065959439	24.334	32.7	14	3

Note: * means APL-16-5 treated Trim-KO HEK293T / control Trim-KO HEK293T

Supplementary Table 6 The transcriptional profile in HEK293T cells treated with APL-16-5

Gene name	Fold change*	log2 fold change	p-value	q-value
FN1	0.739578476	-0.435224856	1.28E-07	8.77E-06
LAMB1	0.968002331	-0.046917574	0.516122473	0.755978939
SLC1A3	0.74418878	-0.426259456	2.20E-06	9.83E-05
GALNT1	1.054993586	0.077234228	0.218937922	0.486386638
PCLAF	1.031367611	0.044558645	0.57204708	0.794132877
PON2	1.16833422	0.224453038	0.047947465	0.197101165
RAB12	1.140804016	0.190050966	0.046638594	0.193419069
FAM117B	1.049313333	0.069445541	0.531576006	0.766628335
GOLM1	0.931058885	-0.103055681	0.06449096	0.235418281
SHCBP1	0.944593419	-0.082234611	0.340316867	0.614591198
TEFM	1.189808057	0.250728853	0.066525094	0.239614387
SDF4	0.962526859	-0.055101295	0.415632531	0.6830825
RIOK2	0.955503851	-0.065666407	0.444099643	0.704384826
RBM24	0.893659664	-0.162202587	0.447848435	0.707890291
KLF16	1.177329352	0.235517964	0.00283096	0.02950327
ETNK1	0.808994667	-0.305797903	1.90E-05	0.000612011
ACOT13	1.031653265	0.044958167	0.596094814	0.808774839
SMYD2	1.064113895	0.089652575	0.37774027	0.649231089
NDUFB11	1.033580557	0.047650837	0.507259193	0.750306405
TMEM9	0.911686883	-0.133389676	0.055705983	0.216699657
WRAP73	1.039747969	0.056233867	0.517174297	0.755978939
MAD2L2	1.008366938	0.012020722	0.875947622	0.953069459

Note: * means APL-16-5 treated HEK293T / control HEK293T

Supplementary Table 7 The selectivity of degradation by APL-16-5 using a multi-omics approach

Gene name	Protein profile of cells treated with APL-16-5		Protein profile in the TRIM25 KO cells treated with APL-16-5		The transcriptional profile of cells treated with APL-16-5	
	log2FoldChange	p-value	log2FoldChange	p-value	log2FoldChange	p-value
FN1	-1.648823997	7.02E-05	1.079224489	0.00057006	-0.435224856	1.28E-07
GALNT1	-0.70181093	0.001527578	0.176195095	0.33929835	0.077234228	0.218937922
PON2	-0.638007114	0.023661548	0.013497992	0.898420518	0.224453038	0.047947465
RAB12	-0.635315518	0.03839378	0.049212535	0.717959892	0.190050966	0.046638594
SHCBP1	-0.658583476	0.020997328	0.497637911	0.000601133	-0.082234611	0.340316867
TEFM	-0.660406499	0.003313502	-0.131787364	0.904167113	0.250728853	0.066525094
RIOK2	-0.723801135	0.003462745	0.275007047	0.023858405	-0.065666407	0.444099643
KLF16	-0.976247864	0.00280039	-0.141143704	0.513164405	0.235517964	0.00283096
ACOT13	-0.781652791	0.011777189	0.014640947	0.85684446	0.044958167	0.596094814
NDUFB11	-0.820616346	0.029472673	-0.479351464	0.39918933	0.047650837	0.507259193
TMEM9	-0.66428809	0.024264699	0.071899932	0.63580078	-0.133389676	0.055705983
WRAP73	-0.674843882	0.003325938	0.307778227	0.206161033	0.056233867	0.517174297
MAD2L2	-0.81400663	0.047251808	-0.250851933	0.065959439	0.012020722	0.875947622
