# Supplementary material for: An anti-influenza A virus microbial metabolite acts by degrading viral endonuclease PA
Source: Nat Commun. 2022 Apr 19;13:2079. doi: 10.1038/s41467-022-29690-x (PMC9019042; doi:10.1038/s41467-022-29690-x)
Supplement: Supplementary file 3 — Reporting Summary [file 41467_2022_29690_MOESM3_ESM.pdf]

nature research

Corresponding author(s): Shan Cen

Last updated by author(s): Feb 11, 2022

Reporting Summary

Nature Research wishes to improve the reproducibility of the work that we publish. This form provides structure for consistency and transparency in reporting. For further information on Nature Research policies, see our Editorial Policies and the Editorial Policy Checklist.

Statistics

For all statistical analyses, confirm that the following items are present in the figure legend, table legend, main text, or Methods section.

n/a	Confirmed	
☐	☒	The exact sample size (*n*) for each experimental group/condition, given as a discrete number and unit of measurement
☐	☒	A statement on whether measurements were taken from distinct samples or whether the same sample was measured repeatedly
☐	☒	The statistical test(s) used AND whether they are one- or two-sided<br>*Only common tests should be described solely by name; describe more complex techniques in the Methods section.*
☒	☐	A description of all covariates tested
☒	☐	A description of any assumptions or corrections, such as tests of normality and adjustment for multiple comparisons
☐	☒	A full description of the statistical parameters including central tendency (e.g. means) or other basic estimates (e.g. regression coefficient) AND variation (e.g. standard deviation) or associated estimates of uncertainty (e.g. confidence intervals)
☐	☒	For null hypothesis testing, the test statistic (e.g. *F*, *t*, *r*) with confidence intervals, effect sizes, degrees of freedom and *P* value noted<br>*Give P values as exact values whenever suitable.*
☒	☐	For Bayesian analysis, information on the choice of priors and Markov chain Monte Carlo settings
☒	☐	For hierarchical and complex designs, identification of the appropriate level for tests and full reporting of outcomes
☒	☐	Estimates of effect sizes (e.g. Cohen's *d*, Pearson's *r*), indicating how they were calculated

Our web collection on statistics for biologists contains articles on many of the points above.

Software and code

Policy information about availability of computer code

Data collection	No custom software was used. Protein blot signals were determined using a Gel Doc XR+ molecular imager (Bio-Rad). QuanStudio Design & Analysis software (version1.5.1) was used to collect qRT-PCR data. Confocal microscopy and image acquisition were performed with PerkinElmer UltraView VoX confocal imaging system. MikroWin 2010 (version 5.17) was used to collect Gluc activity raw data. Octet RED (ForteBio, Inc., CA, USA) was used to measure binding affinity and kinetic profile.
Data analysis	Volocity software(Nikon, version 5.4) was used to analyze confocal microscope images. The Microsoft Excel 2014 and Graphpad Prism (version 8.0) were used for statistics and bar graphs overlaid with dot plots. Image J (version 1.50i) was used to quantify the protein band density. Data analysis on the ForteBio Octet RED instrument was performed using the reference subtraction in the ForteBio data analysis software (version 9.0). MaxQuant software (version 1.3.0.5) was used to processed the resulting MS/MS data. Cufflinks (version 0.8.0) was used to calculate FPKM of each gene.

For manuscripts utilizing custom algorithms or software that are central to the research but not yet described in published literature, software must be made available to editors and reviewers. We strongly encourage code deposition in a community repository (e.g. GitHub). See the Nature Research guidelines for submitting code & software for further information.

Data

Policy information about availability of data

All manuscripts must include a data availability statement. This statement should provide the following information, where applicable:

- Accession codes, unique identifiers, or web links for publicly available datasets
- A list of figures that have associated raw data
- A description of any restrictions on data availability

-All the figures have associated raw data in this paper.

-All relevant data supporting the findings of this study are available within the paper and/or Source data files and Supplementary Information.

Field-specific reporting

Please select the one below that is the best fit for your research. If you are not sure, read the appropriate sections before making your selection.

[x] Life sciences   [ ] Behavioural & social sciences   [ ] Ecological, evolutionary & environmental sciences

For a reference copy of the document with all sections, see nature.com/documents/nr-reporting-summary-flat.pdf

Life sciences study design

All studies must disclose on these points even when the disclosure is negative.

Sample size	Sample size calculation was not performed. We chosed three individual cell lines to observe the antiviral phenotype of APL-16-5. For protein colocalization, the PLA fluorescence signals were quantified from 20 cells. At least three independent biological experimemts were used to perform statistical analysis. Statistical significance between groups was determined using unpaired two-tailed Student's t-tests if without specification.
Data exclusions	No data were excluded.
Replication	Graphs in this manuscript show the mean of at least three independent biological replicates. Replications were described in figure legends.
Randomization	Samples were chosen randomly in animal experiments. Other in vitro experiments were done in individual tissue culture dishes with paired controls to ensure validity.
Blinding	Investigators collecting data of the animal experiment were blinded for the animal groups. For in vitro studies, blinding was not relevant to this study as all the experimental results were obtained by standard cellular or biochemical assays.

Reporting for specific materials, systems and methods

We require information from authors about some types of materials, experimental systems and methods used in many studies. Here, indicate whether each material, system or method listed is relevant to your study. If you are not sure if a list item applies to your research, read the appropriate section before selecting a response.

Materials & experimental systems

n/a	Involved in the study	
[ ]	[x]	Antibodies
[ ]	[x]	Eukaryotic cell lines
[x]	[ ]	Palaeontology and archaeology
[ ]	[x]	Animals and other organisms
[x]	[ ]	Human research participants
[x]	[ ]	Clinical data
[x]	[ ]	Dual use research of concern

Methods

n/a	Involved in the study	
[x]	[ ]	ChIP-seq
[x]	[ ]	Flow cytometry
[x]	[ ]	MRI-based neuroimaging

Antibodies

Antibodies used	Rabbit anti-NP(GTX125989; Genetex), Rabbit anti-PB1(GTX125923; Genetex), Rabbit anti-PB2 (GTX125925; Genetex), Rabbit anti-PA (GTX125932; Genetex), Mouse anti-β-actin(ab8224; Abcam), Mouse anti-core (ab2740; Abcam), Goat anti-Myc (ab9132; Abcam), anti-TRIM25 (610570; BD Biosciences), Anti-HA (SC-7392; Santa Cruz), Anti-SIRT7 (TA326876; OriGene) Goat anti-rabbit IgG horseradish peroxidase (HRP)-linked antibody (ZB-2301), and anti-mouse IgG HRP-linked antibody (ZB-2305) from Beijing Zhongshan Jinqiao Biotechnology, Alexa Fluor-conjugated secondary antibodies (S11223) from Thermo Scientific.
Validation	All the primary antibodies were purchased from commercial sources and validated by the vendor. Validation data are available from the vendor's respective websites.<br>1.Rabbit anti-NP(GTX125989; Genetex) https://www.genetex.cn/Product/Detail/Influenza-A-virus-Nucleoprotein-antibody/GTX125989<br>2. Rabbit anti-PB1(GTX125923; Genetex) https://www.genetex.cn/Product/Detail/Influenza-A-virus-PB1-protein-antibody/GTX125923<br>3. Rabbit anti-PB2 (GTX125925; Genetex) https://www.genetex.cn/Product/Detail/Influenza-A-virus-PB2-protein-antibody/GTX125925<br>4. Rabbit anti-PA(GTX125932; Genetex) https://www.genetex.cn/Product/Detail/Influenza-A-virus-PA-protein-antibody/GTX125932<br>5. Mouse anti-β-actin(ab8224; Abcam) https://www.abcam.cn/beta-actin-antibody-mabcam-8224-loading-control-ab8224.html<br>6. Mouse anti-core (ab2740; Abcam)https://www.abcam.cn/hepatitis-c-virus-core-1b-antibody-c7-50-ab2740.html<br>7. Goat anti-Myc (ab9132; Abcam)https://www.abcam.cn/myc-tag-antibody-ab9132.html

8. Goat anti-TRIM25 (610570; BD Biosciences) https://www.bdbiosciences.com/zh-cn/products/reagents/microscopy-imaging-reagents/immunofluorescence-reagents/purified-mouse-anti-human-efp.610570
9. Mouse anti-HA (SC-7392; Santa Cruz) https://www.scbt.com/p/ha-probe-antibody-f-7?requestFrom=search
10. Rabbit anti-SIRT7 (TA326876; OriGene) https://www.origene.com.cn/catalog/antibodies/primary-antibodies/ta326876/sirt7-rabbit-polyclonal-antibody

Eukaryotic cell lines

Policy information about cell lines

Cell line source(s)	HEK293T (CRL-3216), A549 (CRL-185), MDCK (CRL-34), Vero (CCL-81) and BHK21 (CCL-10) were purchased from American Type Culture Collection (ATCC). Huh7.5.1 cells were provided by Dr. Rongtuan Lin, McGill University.
Authentication	These cell lines were not further authenticated after being received from supplier.
Mycoplasma contamination	We confirm that all cells were tested as mycoplasma negative.
Commonly misidentified lines (See ICLAC register)	No commonly misidentified cell lines were used.

Animals and other organisms

Policy information about studies involving animals; ARRIVE guidelines recommended for reporting animal research

Laboratory animals	Four- to six-week-old female BALB/c mice (Academy of Military Medical Sciences Laboratory, China).
Wild animals	None
Field-collected samples	None
Ethics oversight	All animal experiments were approved by the Institutional Animal Care and Use Committee of the Institute of Medicinal Biotechnology of the Chinese Academy of Medical Sciences. All staff working on animal experiments completed education and training programs according to the standard protocols.

Note that full information on the approval of the study protocol must also be provided in the manuscript.
